# Mechanobiology and survival strategies of circulating tumor cells: a process towards the invasive and metastatic phenotype

**DOI:** 10.3389/fcell.2023.1188499

**Published:** 2023-05-05

**Authors:** Keerthi Kurma, Catherine Alix-Panabières

**Affiliations:** ^1^ Laboratory of Rare Human Circulating Cells (LCCRH), University Medical Centre of Montpellier, Montpellier, France; ^2^ CREEC/CANECEV, MIVEGEC (CREES), University of Montpellier, CNRS, IRD, Montpellier, France; ^3^ European Liquid Biopsy Society (E LBS), Hamburg, Germany

**Keywords:** circulating tumor cells, cancer, mechanobiology, survival, metastasis

## Abstract

Metastatic progression is the deadliest feature of cancer. Cancer cell growth, invasion, intravasation, circulation, arrest/adhesion and extravasation require specific mechanical properties to allow cell survival and the completion of the metastatic cascade. Circulating tumor cells (CTCs) come into contact with the capillary bed during extravasation/intravasation at the beginning of the metastatic cascade. However, CTC mechanobiology and survival strategies in the bloodstream, and specifically in the microcirculation, are not well known. A fraction of CTCs can extravasate and colonize distant areas despite the biomechanical constriction forces that are exerted by the microcirculation and that strongly decrease tumor cell survival. Furthermore, accumulating evidence shows that several CTC adaptations, via molecular factors and interactions with blood components (e.g., immune cells and platelets inside capillaries), may promote metastasis formation. To better understand CTC journey in the microcirculation as part of the metastatic cascade, we reviewed how CTC mechanobiology and interaction with other cell types in the bloodstream help them to survive the harsh conditions in the circulatory system and to metastasize in distant organs.

## 1 Introduction

Cancer is a lethal disease due to its ability to invade and metastasize in distant tissues/organs. This capacity is associated with changes in the tumor microenvironment and with mechanobiological changes in individual cancer cells. Metastatic dissemination (i.e., cancer cell spreading from a primary/initial site to neighboring or distant sites) is a unique feature of cancer cells that typically involves a series of sequential processes, including epithelial-mesenchymal transition (EMT), invasion, intravasation, and extravasation (Fidler, 2003; Gupta and Massagué, 2006; Lambert et al., 2017). Evidence also suggests that cancer cell capacity to detach from the primary tumor and enter the blood or lymphatic vessels as single circulating tumor cells (CTCs) or CTC clusters is significantly influenced by intrinsic and/or extrinsic events in the microcirculation (Varotsos Vrynas et al., 2021). The microcirculation network of small vessels and capillaries establishes a distinct blood microenvironment with extensive and narrow branching that can slow, trap and squeeze CTCs to facilitate their extravasation (Follain et al., 2018). Intriguingly, CTCs might display adaptive mechanisms, such as stiffness and contractility, in response to constricted migration to enhance their survival. These features may also facilitate their extravasation and consequently metastasis formation (Rianna et al., 2020). The first CTC description is attributed to Thomas Ashworth in 1869 (Ashworth, 1869). Since then, CTCs have been considered an important intermediate in the metastatic cascade (Alix-Panabières and Pantel, 2021; Lin et al., 2021; Alix-Panabieres et al., 2022).

Complex biological processes of cancer cells are associated with well-described pathways (e.g., cell adhesion, cytoskeletal remodeling, EMT, and the nucleoskeleton) (Bhadriraju and Hansen, 2002; de las Heras et al., 2013; Ribatti et al., 2020). Conversely, the molecular mechanisms that regulate CTC mechanics in the microcirculation are rather elusive. Moreover, recent studies showed that mechanical changes in cancer cells, such as the dynamic regulation of cell stiffness and contractility, are common phenotypic events in cancer development, progression and metastasis (Xu et al., 2012; Gensbittel et al., 2021). In liquid biopsy (a powerful non-invasive tool for the prognosis, treatment selection, and monitoring of several cancer types) (Keller and Pantel, 2019; Alix-Panabières and Pantel, 2021), the quantification of CTC deformability (i.e., the cell capacity to change its shape under load in the microcirculation) is used to obtain the algorithmic-based objective identification of metastatic cancer cells in different body fluids (e.g., blood) (Tse et al., 2013; Dhar et al., 2016; Yu et al., 2022).

However, the specific sequence and molecular networks underlying the changes of CTC mechanotype during the metastatic cascade are not well defined. Yet, studying CTC mechanotypes and the associated molecular events may pave the way to cancer mechanobiology. Very few studies investigated the cellular and molecular mechanisms underlying CTC adaptation and survival in blood, particularly in capillaries. In this review, we discuss the effects of the biomechanical forces experienced by CTCs in the blood circulation and capillaries and outline CTC adaption and survival strategies towards an invasive and metastatic phenotype in this confined environment. Better understanding CTC mechanical properties and how they can be perturbed will contribute to improve *i)* liquid biopsy tests by increasing CTC detection accuracy, and *ii)* the design of personalized therapeutics to stop tumor cell dissemination and cancer progression.

## 2 Cancer metastases and circulating tumor cells

**FIGURE 1:**
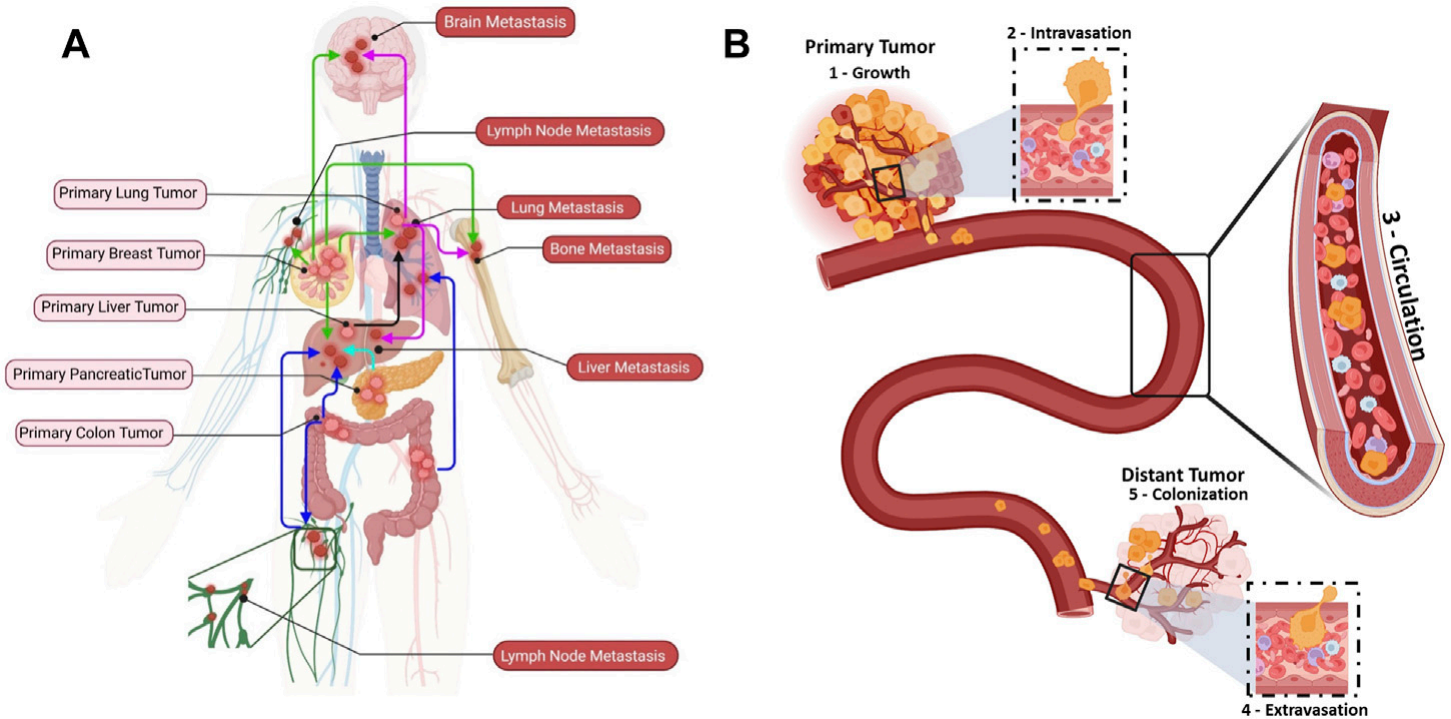
Metastatic dissemination of circulating tumor cells (CTCs) to distant organs. **(A)** Examples of cancer types that metastasize to distant organs: breast cancer (green), lung cancer (purple), liver cancer (black), pancreatic cancer (aqua), and colon cancer (blue). **(B)** The different metastatic cascade steps: **(1)** Growth: uncontrolled proliferation and growth of a primary tumor in a healthy tissue. **(2)** Intravasation: cancer cells undergo substantial changes to endure the physical interactions and mechanical forces in the tissue and intravasate the bloodstream through endothelial cells. **(3)** Circulation: once in the bloodstream, cancer cells become CTCs (single CTCs or CTC clusters). CTC clusters are identified as micro-emboli in advanced cancer stage (*homotypic clusters* composed of CTCs only; *heterotypic clusters* composed of CTCs and other cell types, such as immune cells, cancer-associated fibroblasts, platelet). Most CTCs will die within 2 h, but a subset of metastasis-competent CTCs will survive and adapt to extremes conditions to reach the right place where they extravasate. **(4)** Extravasation: CTCs extravasate at specific distant organs. ***(5)*** Colonization: CTCs that arrive at a distant organ and find good conditions to interact with the extracellular matrix (seed and soil concept) will colonize and form metastatic lesions.

Cancer metastasis has been studied for more than one century, but it remains a poorly understood biological process (Figure 1A). In 1889, Stephen Paget proposed the “seed and soil” hypothesis, according to which the crosstalk between cancer and the organ special microenvironment is crucial for cancer metastasis (Paget, 1989). The “metastatic cascade” comprises distinct sequential steps leading to secondary foci or to the colonization of distant organs/tissues (Figure 1B) (Fidler, 2003). Cancer is initiated by uncontrolled cell proliferation and growth of the primary solid tumor in a healthy tissue/organ that undergoes mutations and epigenetic alterations (Figure 1B: Growth) (Vicente-Dueñas et al., 2018). A favorable microenvironment is created for the initial cancer growth, notably through neovascularization, an inflammatory-like immune response, and extracellular matrix (ECM) remodeling (Whiteside, 2006; Stacker et al., 2014; He et al., 2022). Simultaneously, some primary tumor cells acquire stem cell features, such as self-renewal and the ability to differentiate into multiple cell types, by undergoing EMT. EMT leads to downregulation of genes that encode epithelial junction proteins, and consequently to disruption of adherent junctions, desmosomes and tight junctions and to loss of apical-basal polarity (Yang et al., 2020). These changes in epithelial cell junctions affect many different downstream pathways that further promote invasion (Yeung and Yang, 2017). Moreover, the expression of non-epithelial cadherin and cell surface proteins, which are critical for cell migration, is increased (Loh et al., 2019). As the cytokeratin network anchored to desmosomes is disrupted, the cortical actin cytoskeleton is reorganized into an intracellular and basal network of intermediate filaments. The development of novel membrane protrusions, such as invadopodia for matrix degradation, is facilitated by the shift to the mesenchymal state that also promotes cell motility. Additionally, actin stress filaments promote and strengthen cell contractility (Yeung and Yang, 2017). Finally, the increased number of cell protrusions and the loss of cell-cell and cell-matrix adhesion, the secretion of metalloproteinases that digest the ECM, the motility and invasive behavior allow tumor cells to invade the stroma and then to intravasate to become CTCs (Figure 1B: Intravasation) (Gritsenko et al., 2017; Sznurkowska and Aceto, 2022).

Intriguingly, cancer cell (single cells or in clusters) intravasation can be facilitated by immune cells in the tumor microenvironment (Aceto et al., 2014). However, a distant metastasis develops only when tumor cells can spread throughout the blood and/or lymphatic vessels (Figure 1B: Circulation) (Brown et al., 2018; Pereira et al., 2018). In the circulation, CTCs “swim” in a very harsh environment and they will eventually arrest and adhere to the walls of vessels in distant tissues or organs where they can extravasate. Before reaching this step, they must survive the aggression by immune cells and endure the blood flow forces and adhesion forces (Osmani et al., 2019). CTCs extravasate and then initiate a distant metastasis once they find a favorable host microenvironment, known as the pre-metastatic niche (PMN). A PMN is formed through a multi-step process initiated by local (host) changes through the involvement of secretory factors and extracellular vesicles that induce vascular leakage, ECM and stroma remodeling, and immunosuppression (Peinado et al., 2017). Then, this favorable environment supports the overt colonization (i.e., tumor cell seeding) via cell-ECM interactions that supply an anchorage point for seeding, and also activate survival and proliferative signaling programs transduced through integrin complexes and their associated downstream signaling. For instance, adhesion receptors of the integrin family (e.g., integrins αvβ3, β1 and β4) are involved in CTC adhesion to endothelial cells during extravasation (Reymond et al., 2012; Barthel et al., 2013). Similarly, *in vivo* studies using zebrafish cancer cell xenograft models showed that the glycoprotein CD44 and the integrins αvβ3 and α5β1 are required for weak (CD44 and αvβ3) and strong (α5β1) interactions between CTCs and endothelial cells (Osmani et al., 2019). Moreover, the integrin α4β1, which is expressed by cancer cells, can serve as an alternative ligand for vascular cellular adhesion molecule 1 (VCAM-1 or CD106) to mediate the firm adhesion of cancer cells to the endothelium (Strell and Entschladen, 2008). In addition, neuropilin-2, expressed by CTCs, interacts with integrins, expressed on the endothelial cell surface, to promote cell attachment (Cao et al., 2013). Integrins are also found on tumor-derived extracellular vesicles and can attach to ECM molecules in organ-specific PMNs, promoting organotropic metastases. Hoshino *et al.* showed that tumor-associated exosomes present cancer-specific integrin profiles associated with the formation of a PMN in specific organs, for instance integrin αvβ5 and liver, α6β4, and α6β1 and lung (i.e., cancer cell organotropism) (Hoshino et al., 2015). (Figure 1B: Extravasation). On the other hand, CTCs may remain in a dormant state until their new environment changes and allows them to thrive and to form distant metastases (Aguirre-Ghiso, 2007; Hedley and Chambers, 2009; Vishnoi et al., 2015; Boral et al., 2020).

During these metastatic cascade steps, tumor cells and CTCs are subjected to a variety of external mechanical stresses that affect their survival and progression and that may lead to the acquisition of specific mechanical properties (Wirtz et al., 2011; Mohammadi and Sahai, 2018). For instance, during the early stages of tumor growth, tumor cells experience increasing levels of compressive stress due to the imbalance in fluid entry and exit into the interstitial space and the microenvironment pushing back the expanding tumor (Jain et al., 2014). Moreover, cancer tissues are generally stiffer than healthy tissues due to tumor volume expansion, increased compressive stress, and ECM stiffening (Wirtz et al., 2011; Chin et al., 2016; Nia et al., 2016). In addition, due to compressive stress, the confining primary tumor environment also favors collective cancer cell invasion (to give rise to CTC clusters), which is recognized as a necessary step for distant metastases (Haeger et al., 2014). Intravasation can occur early and throughout the tumor progression, and concerns even non-transformed epithelial cells. Moreover, it has been shown that intravasation occurs both through active cancer cell invasion of blood vessels and passive cancer cell shedding in the circulation (Bockhorn et al., 2007). These processes are triggered by mechanical stress on the tumor and/or disorganization within the tumor microenvironment.

CTCs remain enigmatic in many ways, partly, due to difficulties in studying these rare cells. Once CTCs have entered the circulation, most available evidences indicate that they stay there only very briefly, although measuring CTC half-life in the circulation has been quite challenging (Meng et al., 2004; Stott et al., 2010; Aceto et al., 2014). CTC survival, circulation in the bloodstream, their capacity to colonize distant organs, and how the circulation microenvironment influences CTC biology are open and actively investigated questions. Additionally, after stopping in the microvasculature, CTCs normally interact with the endothelium to begin extravasation (Follain et al., 2018). Our knowledge of the metastatic cascade can be improved by understanding how CTCs behave in capillaries and how they can adapt and acquire a more invasive phenotype. These topics are briefly discussed in the next section.

## 3 Mechanobiology of CTCs in the circulating system

**FIGURE 2:**
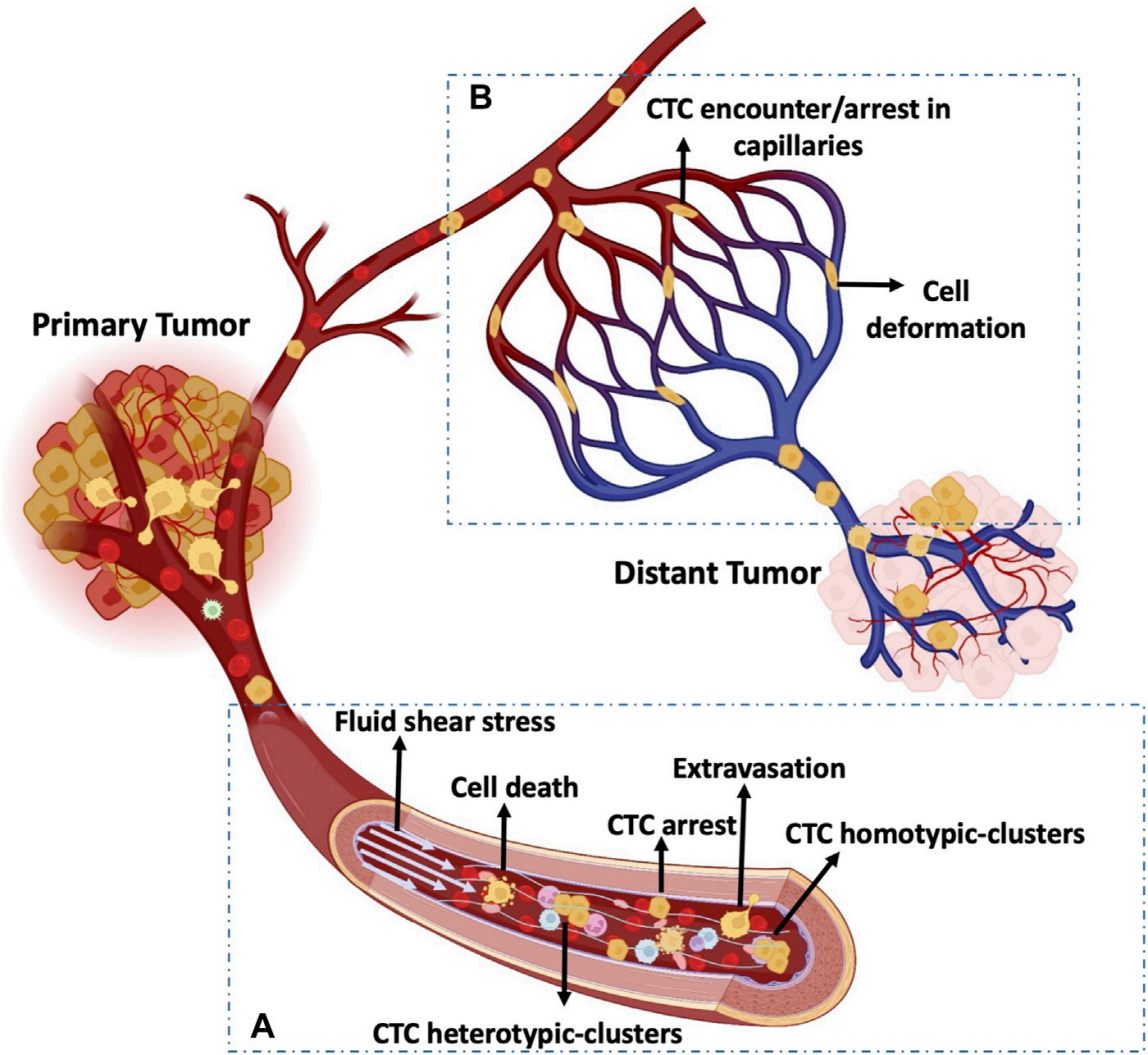
Mechanobiology of CTCs in the circulating system. **(A)** CTCs experience blood fluid shear stress. In the circulation, single CTCs and homo/hetero-typic CTC clusters are exposed to various hemodynamic shear stresses and may arrest and extravasate, or break and die. **(B)** CTC arrest and deformability in the capillary bed. CTCs stop in the capillary bed and undergo phenotypic changes (e.g., stiffness) due to cell deformation induced by biomechanical forces.

The circulation is a remarkable, highly evolved system for the efficient transport of blood cells, gasses, nutrients, and hormones that provides nourishment to tissues and organs and contributes the organism defense, homeostasis, and growth. Cancer cells experience mechanical stress in the primary tumor, and also in the circulation after intravasation (Follain et al., 2020). Escape from the immediate tumor vasculature may be influenced by spatial and temporal heterogeneity in tumor blood flow patterns (Jain, 2005; Kamoun et al., 2010). Once cancer cells enter the systemic circulation and become CTCs, they are in a very harsh and unknown environment, exposed to completely different mechanical stresses due to the hemodynamic forces that constrict blood vessels (Figure 2). This is one of the reasons of CTC short half-life (Wirtz et al., 2011; Labelle and Hynes, 2012; Strilic and Offermanns, 2017). Moreover, CTCs are affected by other factors, such as blood pressure and flow patterns of the organ and also cell deformability and adhesion, enabling them to arrest in the capillary bed. CTCs must overcome all these different stresses.

When CTCs reach transport channels, they experience hemodynamic shear forces because they must flow at the blood velocity, which ranges between 0.5 and 1.5 mm/s in capillaries and reaches 120 cm/s in the aorta (Table 1) (Ivanov et al., 1981; Gisvold and Brubakk, 1982). During circulation, CTCs may undergo cell-cycle arrest or damage due to high fluid shear stress (FSS) (Figure 2A) (Regmi et al., 2017). Few CTCs and/or CTC clusters may escape FSS effects in capillaries and may stop and adhere to the endothelium. Importantly, once CTCs are released in the microcirculation, the first challenge is the size restriction to which they must adapt in few seconds. Indeed, cancer cells from solid tumors are usually larger (15–20 µm) than capillaries (3–12 µm), and the microcirculation acts as a CTC filter because most CTCs are arrested transiently in the first microvascular bed (Fidler, 1970; Luzzi et al., 1998; Cameron et al., 2000; Krog and Henry, 2018; Varotsos Vrynas et al., 2021). It is also important to consider the temporal dimension of FSS exposure. Indeed, CTCs may only freely circulate for seconds and most of the time they could be trapped in the microcirculation (Krog and Henry, 2018). However, the amounts of time spent by CTCs in free circulation and trapped in capillaries are still unknown. Certainly, some CTCs can escape from the microcirculation because they are detected in blood samples from the patients’ arm veins. Thus, cancer cells are deformable and motile and these properties may contribute to their ability to negotiate the microcirculation barrier and colonize distant sites (Figure 2B). It can be hypothesized that only deformable CTCs are detected in the circulation. Indeed, mechanical deformability has been investigated as a potential CTC detection technique (cancer mechanobiology) (Kumar et al., 2022). However, only extremely sophisticated and specialized techniques, such as single-cell atomic force microscopy, tiny needle aspiration, single-cell liquid biopsy, can be used to perform such mechanical assessments (Kumar et al., 2022). Moreover, some of the existing technologies for CTC enrichment and detection, such as ISET^TM^ (Hofman et al., 2011) and Parsotix^TM^ (Xu et al., 2015), are based on CTC size and deformability. However, these approaches present some limitations, such false positive detection and low specificity.

**TABLE 1:** Body fluid systems and their velocities and fluid shear stresses (Cao et al., 2016; Follain et al., 2020).

Fluid system	Fluid velocity (flow rate)	Fluid shear stress
Interstitial fluid	0.001–0.004 mm/s	0.1–1 dyn/cm^2^
Lymphatic system	0.02–1 mm/s	0.64–12 dyn/cm^2^
Arterial flow	100–500 mm/s	4–30 dyn/cm^2^
Venous flow	1–200 mm/s	1–4 dyn/cm^2^
Capillary bed	0.01–1.5 mm/s	10–20 dyn/cm^2^
Heart flow (valve leaflets)	1.26 m/s	100 dyn/cm^2^

CTC clusters are aggregates that may include only few CTCs (homotypic clusters) or CTCs and stromal/immune cells (heterotypic clusters). CTC clusters have higher metastatic potential than single CTCs due to their larger size and a slower travelling velocity (Fidler, 1973; Aceto et al., 2014). Indeed, the lower travelling velocity of CTC clusters increases the chance of margination and attachment to the vascular wall, even in vessels with diameters too large to intercept clusters (King et al., 2015). Moreover, Au *et al* showed that over 90% of CTC clusters could pass through capillary-sized vessels by rapidly and reversibly reorganizing into single-file chain-like geometries that substantially reduce their hydrodynamic resistances (Au et al., 2016). Interestingly, and contrary to the assumption that CTC clusters cannot transit through narrow vessel, Au *et al.* findings demonstrated that the CTC cluster shape is highly plastic and that its cells can easily reorganize again into a sphere-like cluster after having traversed the capillaries (Au et al., 2016). Therefore, despite the intense mechanical deformation observed in CTC clusters when moving through capillaries, the cooperation between cells within the cluster might explain the cluster survival advantage compared with single CTCs (Au et al., 2016; Chen et al., 2018; Giuliano et al., 2018; Genna et al., 2020). The different cells present in heterotypic CTC clusters may protect against FSS and immune attacks by producing a coating shield (Tesfamariam, 2016; Yang et al., 2020). For example, Szczerba *et al.* demonstrated that the crosstalk between cancer cells and neutrophils within the cluster promotes CTC extravasation (Szczerba et al., 2019).

CTC arrest in the blood circulation can take two forms: i) active adhesion to vessel walls and ii) occlusion if the vessel is topographically disordered and/or narrow. Follain *et al.* demonstrated *in vivo* that low blood flow profiles can promote cell arrest without the need of physical occlusion, suggesting that active adhesion of CTCs to the endothelium is required for successful extravasation (Follain et al., 2018). However, the frequency of escape from the first capillary where they intravasate and whether this is an inactive or active process are not clear.

### 3.1 CTCs and fluid shear stress

Cancer cells are exposed to FSS even before entering the circulation in the form of interstitial fluid flows that produce cell surface fluid shear stress of 0.1–10 dyn/cm^2^ (Swartz and Lund, 2012). When cancer cells enter the bloodstream, they are exposed to a completely new environment of greatly varying FSS levels (Table 1) that might be lethal for most of them (Follain et al., 2020). Although CTCs can survive exposures to extreme levels of FSS for brief periods, compared with normal epithelial cells (Barnes et al., 2012), high FSS might result in mechanical stress that can cause CTC rupture and death. Conversely, moderate shear forces favor CTC intravascular arrest and extravasation (Regmi et al., 2017; Xin et al., 2019). Regmi and others demonstrated an increase in breast CTC destruction using a microfluidic circulatory system that mimics the FSS achieved during intense exercise. In their study, necrosis occurred after 4 h and apoptosis after 16–24 h of flow (Regmi et al., 2017). Additionally, FSS generated by the frictional pressures imposed on CTCs when blood flow travels tangentially over the CTC surface can trigger mechanosensitive ion channels, such as Piezo1 and Piezo2 (Martial, 2016). For instance, it has been demonstrated that under circulatory FSS, Piezo1 activation increases the susceptibility of colorectal, prostate, and breast cancer cells to the apoptosis-inducer TRAIL (Hope et al., 2019). Moreover, during cancer cells/CTCs migration through restricted areas, Piezo1 is activated due to membrane stretching, raising the intracellular Ca^2+^ content. Piezo1 channels, in conjunction with myosin II, promote malignant cell motility in constrained areas, particularly facilitating efficient migration through narrow channels (Hung et al., 2016).

To determine whether CTC clusters behave like single CTCs upon exposure to FSS, Marella and others created a multichannel microfluidic device to reproduce the various FSS levels that characterize the human circulatory system (capillaries, veins, and arteries) and demonstrated cluster disaggregation at high FSS (Marrella et al., 2021). On the other hand, FSS may lead to CTC arrest, adhesion, and rapid extravasation to limit the risk of being destroyed. An *in vitro* study showed that CTCs are more resistant to FSS than expected because of a transient response triggered by plasma membrane damage that relies on extracellular calcium and actin cytoskeletal dynamics (Barnes et al., 2012). Moreover, for CTC survival, lamin A/C is essential to maintain cell integrity and to repair the plasma membrane damage caused by FSS (Mitchell et al., 2015). Upon exposure to FSS, CTC clusters develop their own survival strategies, such as the generation of stable cell aggregates resistant to anoikis (i.e., apoptosis induced by inadequate or inappropriate cell-matrix interactions) in an E-cadherin-dependent manner (Maeshiro et al., 2021).

Studies on cell survival to FSS showed that the actomyosin cytoskeleton has critical but contradictory functions. For instance, in an *in vitro* study, FSS-surviving CTCs showed decreased actomyosin activity, resulting in lower cell stiffness and also increased chemotherapy resistance (Xin et al., 2019). Conversely, another study found that actomyosin activity and cell stiffness enhanced tumor cell survival during FSS and that CTC clusters increased their stiffness in response to FSS via actomyosin activity, which is mediated by Ras Homologous A (RhoA) and non-muscle myosin II activity (Moose et al., 2020). Interestingly, CTC interaction with other cells, such as platelets (Tesfamariam, 2016; Ward et al., 2021), neutrophils (Chen et al., 2018; Szczerba et al., 2019), macrophages and cancer-associated fibroblasts (CAFs), allows them to resist FSS damage in the blood circulation. For example, in clusters with CTCs, CAFs increase resistance to FSS via intercellular contacts and soluble actors and by protecting CTC capacity to proliferate (Ortiz-Otero et al., 2020). Although FSS effects on CTC mechanics are not fully understood, a recent study suggested that FSS-induced EMT may play a role in improving CTC survival and metastatic potential (Choi et al., 2019; Yu et al., 2021). Thus, the mechanotransduction process that allows CTCs to survive to FSS may offer a unique therapeutic approach for CTC elimination and metastasis prevention. However, no study has brought any direct evidence that the association with other cell types allows CTCs to resist mechanical damage.

## 4 Determinants of cancer cells/CTC mechanical properties

CTCs experience mechanical forces and stress that affect their shape, migration and fate once they are in the microcirculation or arrested in vascular capillaries during intravasation and extravasation. Cancer cell mechanics are determined by major components, particularly the nuclear envelope, the cytoskeleton and the ECM. The ultimate consequence of cancer cell mechanical abnormalities is an altered potential for invasion and metastasis.

### 4.1 Nuclear envelope

In the circulation and microcirculation, the CTC nucleus experiences deformations that facilitate cell displacement in response to capillary-induced constriction. It has been demonstrated that nuclear deformation is the primary steric barrier to cell migration in confined spaces (Lautscham et al., 2015; Kamyabi and Vanapalli, 2016; Xia et al., 2018). The nucleus, which is the largest cell organelle, actively engages with the mechanical environment in addition to serve as a load-bearing organelle (Caille et al., 2002). The nucleus is typically stiffer than the cytoplasm (Dahl et al., 2008) and its regulation can ensure the successful transit of CTCs through the capillary beds to reach an optimal microenvironment where they can arrest and extravasate (Lammerding, 2011). The major components of nucleus mechanics include the nuclear membrane, membrane proteins, nuclear lamina, chromatin and intranuclear proteins. In cancer cell mechanobiology, the nuclear membrane structure and the protein network (lamin) underlying the inner nuclear membrane, known as the nuclear envelope, have been intensively explored (Chow et al., 2012). The nuclear membrane is made of two lipid bilayers (the outer nuclear membrane and inner nuclear membrane). The inner membrane is covered by the nuclear lamina with two lamin types (type A: lamin A and lamin C; and type B: lamin B) (Peter and Stick, 2012). Lamin A and C are encoded by *LMNA* and provide the nucleus viscous stiffness (Donnaloja et al., 2020), whereas B-type lamins confer the nucleus elasticity (Vahabikashi et al., 2022). Besides providing structural support to the nucleus, the nuclear lamina also regulates its shape and size. For instance, cancer cells that migrate through small pores (<6 μm^2^) show altered lamin expression (Denais et al., 2016). Lamins also enhance the nucleoskeleton capacity to stand mechanical stress by interacting with chromatin and several intranuclear proteins, such as nuclear actin, spectrin and myosin (Simon and Wilson, 2013). Lamin A plays an essential role in mechanosensitive differentiation. Low levels of lamin A and C in cancer cells reduce cell stiffness, leading to more invasive phenotypes (Harada et al., 2014; Dubik and Mai, 2020; Bell et al., 2022). Therefore, it could be hypothesized that CTCs might successfully intravasate/extravasate across the constrained environment of vascular capillaries by modulating nuclear stiffness through lamins. Low expression of lamins A/C may favor CTC migration through confined environments, but it can also result in decreased resistance to FSS, leaving the nucleus unprotected during migration and decreasing CTC viability. Reduced lamin levels reduce stiffness and promote invasion (Mitchell et al., 2015). In agreement, other studies demonstrated that low lamin A/C levels are correlated with worse prognosis and the development of distant metastases in breast (Alhudiri et al., 2019), gastric (Wu et al., 2009), lung (Kaspi et al., 2017) and colon cancer (Belt et al., 2011). The nucleus is physically connected with the surrounding cytoskeleton by proteins. This connection is facilitated by linker of the nucleoskeleton and cytoskeleton (LINC) complexes that are multicomponent structures spanning through the nuclear envelope. The interactions between LINC complexes and cytoskeleton are also crucial for cell migration in pathological situations, such as cancer metastasis (Wirtz et al., 2011). More studies are needed to evaluate CTC arrest and extravasation capacities in relation to LMNA expression to better understand the role of CTC stiffness regulation in the metastatic cascade.

### 4.2 Cytoskeleton

The cytoskeleton is an intracellular network of filaments and tubules that extends from the nucleus to the plasma membrane. All cell types have three types of cytoskeletal proteins: microfilament actin, intermediate filaments, and microtubules. Mechanical forces affect all three cytoskeleton components that display different elastic properties and contribute to stabilize the cell morphological and architectural structures. Additionally, these proteins play a significant role in cancer development and progression because they actively perceive and respond to the cell environment, shape the cell, and alter the cell functions (Hall, 2009; Fife et al., 2014; Aseervatham, 2020). For instance, hypoxia-induced signaling can promote EMT that results in a range of cytoskeletal alterations, including the production of chemotherapy-resistant tubulin isoforms and the intermediate filament vimentin (Huang et al., 2019; Datta et al., 2021). Likewise, when a mechanical force is applied to cancer cells, actin filaments act a mechanosensor that senses the mechanical forces (Jégou et al., 2013). However, the alteration of actin remodeling is a dynamic, two-way process. Actin polymerization/depolymerization is finely regulated by a series of actin signaling proteins that are also part of important oncogenic signal transduction pathways (Suresh and Diaz, 2021). Moreover, the actomyosin cytoskeleton, one of the main cytoskeleton components, contributes to membrane deformation via its interaction with the cell membrane and the cytoskeleton (Farsad and De Camilli, 2003). The actomyosin cortex is in charge of the cell cortical tension. Cortical tension restricts cell deformation and is involved in membrane bulges and cell contractions. It also contributes to cell stiffness, tension levels, and breakages in the cortex. As a result, it is an active participant in the cell mechanics and offers resistance to cell deformation upon exposure to small strains (Paluch et al., 2005; Chugh and Paluch, 2018; Koenderink and Paluch, 2018). Intermediate filaments are important for resistance to cell deformation in response to large strains and operate as a safety net against severe distortion that would damage the cell (Charrier and Janmey, 2016). Therefore, the cytoskeleton is crucial for the cell stiffness and capacity to passively deform, and also for the general cell shape actively adopted during various activities (Bhadriraju and Hansen, 2002). The cytoskeleton also places the cell organelles inside its network of filaments, providing a direct connection between cell shape/deformation and important organelle repositioning during the metastatic cascade. Therefore, the cytoskeleton, which participates in both active and passive mechanical processes, may be seen as a key player in CTC mechanics for survival adaptations in the circulation.

### 4.3 Extracellular matrix

The ECM contains many cells and non-cell components involved in cell-cell and cell-matrix interactions, and is a highly dynamic and organized meshwork (Frantz et al., 2010). The ECM is made of four primary fibrous proteins (collagen, proteoglycans, laminin, and fibronectin) and also growth factors, minerals, and water. It provides critical signals to preserve the tissue architecture, polarity, and homeostasis and to regulate cell growth and apoptosis. During cancer progression, alterations in tumor cell-ECM interactions have a significant role in regulating tumor development, malignant transformation, invasion, and metastasis, as well as treatment resistance (Winkler et al., 2020). The impact of ECM remodeling at each phase of metastasis development, from cancer survival in the circulation to the establishment of the pre-metastatic and metastatic niches, has been extensively studied in the past 10 years (Winkler et al., 2020).

The interstitial matrix and basement membrane are two major and distinct ECM types in terms of function, composition, and location. Remodeling of the interstitial ECM in cancer causes a wide range of biophysical and biochemical alterations that affect cell signaling, ECM stiffness, cell migration, and tumor growth (Egeblad et al., 2010). Conversely, the basement membrane is a more stable, sheet-like, dense structure that lines the basal surface and separates tissues into different and well organized compartments (Yurchenco, 2011). Cancer cells must remodel the basement membrane to infiltrate a stromal tissue (Chang and Chaudhuri, 2019). Various ECM remodeling events support the migration and invasion of tumor cells that eventually will enter the circulation. For instance, single-cell RNA-sequencing of pancreatic cancer-derived CTCs revealed that CTCs in the blood upregulate the expression of common stroma-derived ECM proteins (Ting et al., 2014). Moreover, transmembrane surface molecules, such as integrins and some glycoproteins, can sense the surrounding ECM fibers and function as cellular mechano-chemical sensors. For example, in the blood circulation, CTCs bypass anoikis by secreting fibronectin to reinforce integrin-dependent adhesion survival signaling (Barbazán et al., 2012). Furthermore, via heterotypic contacts with non-tumor cell types (e.g., platelets and macrophages), CTCs can promote pro-survival adhesion signaling. CTC-associated ECM can improve autocrine survival signaling in CTCs, shield them from immune cell clearance, as observed for platelets surrounding CTCs, and promote the development of CTC clusters for effective metastatic colonization (Winkler et al., 2020). CTCs arriving in secondary organs typically initiate and drive ECM remodeling. For example, breast cancer cells metastasizing to the lung produce their own tenascin C that promotes survival and macro-metastasis outgrowth via the NOTCH and WNT stem cell pathways (Oskarsson et al., 2011). Overall, deciphering the processes linking ECM to CTC survival is crucial for understanding CTC adaptation strategies.

## 5 Signaling pathways activated in CTCs by mechanical cues

**FIGURE 3:**
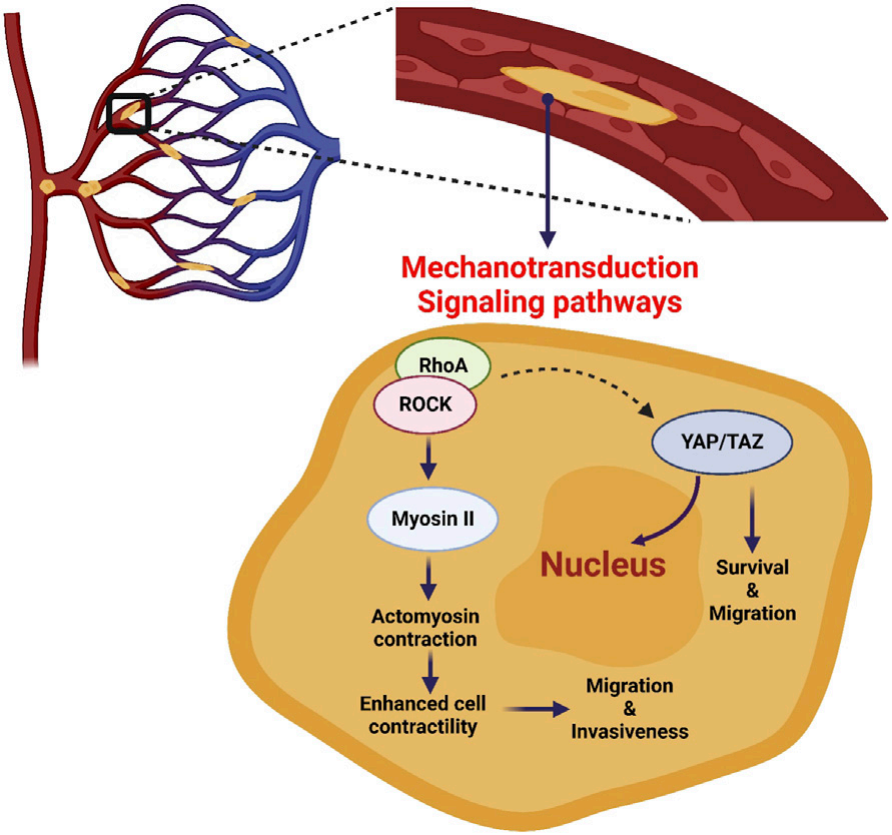
Mechanotransduction signaling pathways of CTCs in capillaries. CTCs experience severe cell deformation during their transit and/or stop in capillaries. This can affect the mechanotransduction of signaling pathways, such as RhoA-ROCK and YAP/TAZ, resulting in increased cell invasiveness, survival and migration.

During extravasation, CTCs need to adapt and modify their mechanical properties in response to mechanical and chemical stimuli. This mechanotransduction process leads to the induction of different signaling pathways that allow CTCs to adhere and migrate through the vascular endothelium. CTCs, like leukocytes, establish various selectin- and integrin-mediated interactions with the endothelial cells that line the capillary walls. For this, CTCs require actin cytoskeleton remodeling, which is centrally regulated by Rho GTPase signaling networks (Reymond et al., 2013; Rodenburg and van Buul, 2021). Rho GTPases are a family that includes twenty small signaling G-proteins. They are also part of the Ras GTPase superfamily that has a central role in cancer (Wennerberg et al., 2005; Heasman and Ridley, 2008). Moreover, mechanical squeezing of CTCs in capillaries increases the activity of RhoA, resulting in the recruitment and activation of Rho-associated protein kinase (ROCK) (Huang et al., 2018). ROCK activation increases the activity of many proteins, including myosin II that binds to actin, leading to the contraction of the actomyosin networks and then to cell contractility increase (Moose et al., 2020). Huang *et al.* demonstrated that when CTCs are confined in capillaries, the RhoA-ROCK-myosin II axis is activated, thus enhancing contractility and resulting in CTC transition from an elongated to a spherical shape (Huang et al., 2018). RhoA-ROCK-myosin II activity appears to be essential for the morphological changes that occur in CTCs during arrest, leading to extravasation (Perea Paizal et al., 2021). The RhoA pathway also seems to be exploited by CTCs to overcome the limitation imposed by FSS and lack of stable arrest (Figure 3) (Huang et al., 2018). However, it is still unknown to what extent the observed RhoA activation in the microcirculation is the result of exposure to FSS in the free circulation *versus* mechanical squeezing in capillaries, an open question for future studies.

Human cancers frequently display hyperactivation of the Hippo signaling pathway and its downstream transcriptional coactivators Yes-associated protein 1 (YAP1) and Transcriptional activator with PDZ-binding motif (TAZ), in response to a variety of stimuli, including mechanical cues (Dupont et al., 2011; Panciera et al., 2017; Martinez et al., 2019). Moreover, through activating transcription factors (e.g., YAP/TAZ or SRF), RhoA induction can have direct effects on the cytoskeleton, cell-ECM adhesion, and cell shape determination (Figure 3) (Cai et al., 2021). For example, Lee *et al.* found that FSS in the lymphatic vasculature (0.05 dyn/cm^2^) promotes YAP1/TAZ translocation to the nucleus, leading to cell motility changes through activation of the ROCK-LIMK-YAP1 signaling axis (Lee et al., 2017). In another study, the same FSS level increased cell division by increasing TAZ expression and promoting its nuclear translocation (Lee et al., 2018). YAP/TAZ nuclear translocation can be induced also independently of RhoA activation by forces applied directly to the nucleus or the cytoplasm. Using atomic force microscopy, Elosegui-Artola *et al.* showed that a force applied to the nucleus was enough to induce YAP nuclear translocation by reducing mechanical restriction in nuclear pores (Elosegui-Artola et al., 2017). Overall, these studies suggest that during CTC transit and/or arrest in capillaries, signaling pathways may be triggered by CTC deformation to enhance their metastatic potential.

## 6 CTCs and hetero-clusters in the circulating system

**FIGURE 4:**
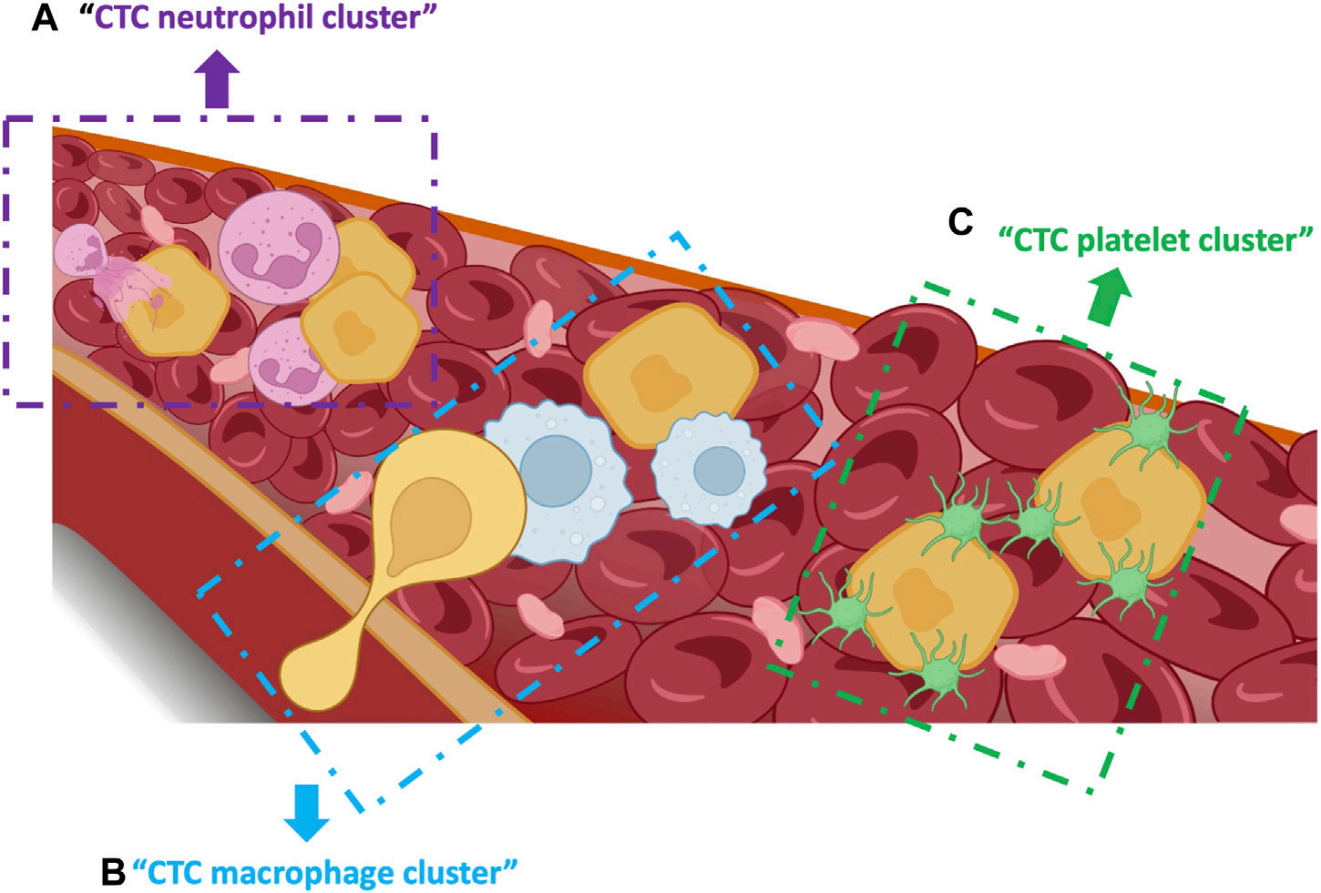
Single CTCs and heterotypic clusters in the bloodstream. **(A)** CTC-neutrophil clusters enhance proliferation via crosstalk of cytokines. **(B)** CTC-macrophage clusters promote CTC extravasation and dissemination and confer resistance to shear stress. **(C)** CTC-platelet clusters promote CTC invasion, survival and metastasis as well epithelial-mesenchymal transition by secreting TGF-beta.

CTCs in the circulation interact with complex fluid ecosystems (blood and lymph) that are composed of different cell types. Despite the harsh survival conditions in the circulation, some CTCs will survive and contribute to metastasis in secondary sites. CTCs in the circulation can be found as heterotypic clusters with white blood cells, particularly CTC-neutrophil clusters. Szczerba *et al.*, showed that the direct interaction of CTCs with neutrophils leads to the upregulation of some neutrophil genes, such as *ARG1, CXCL1, CXCL2, CXCL10, CCL2, CXCR2* and *VEGFA*, resulting also in transcriptomic changes in CTCs and activation of the essential pathways that control cell cycle and DNA replication (Szczerba et al., 2019). Neutrophils in CTC clusters express adhesion factors, such as vascular cell adhesion protein 1 (VCAM-1) and intracellular adhesion molecule 1 receptor (ICAM-1). Moreover, the close interaction between CTCs and neutrophils promotes the activation of proliferative pathways in CTCs via neutrophil cytokines, such as IL-6 and IL-1β (Figure 4A) (Yang et al., 2005; Guo and Oliver, 2019; Iriondo and Yu, 2019; Taftaf et al., 2021). The release of neutrophil extracellular traps (NETs) into the extracellular space also can protect CTCs, via β1-integrin, from shear stress and immune cell-mediated cytotoxicity in the blood (Najmeh et al., 2017).

Tumor-associated macrophages (TAMs) stimulate the subsequent steps: CTC extravasation and dissemination (Figure 4B) (Doak et al., 2018). Hamilton *et al.* co-cultured peripheral blood mononuclear cells with small-cell lung cancer-derived CTC lines to investigate the interactions between CTCs and macrophages. They observed that CTCs can stimulate monocyte differentiation into TAMs, which then release mediators to promote leukocyte recruitment, migration and invasion (Hamilton et al., 2016; Hamilton and Rath, 2017). In another study in colorectal cancer, the feedback loop between TAMs and cancer cells was essential for CTC EMT and intravasation into the bloodstream (Wei et al., 2019). Moreover, TAMs seem to promote CTC acquisition of mechanical adhesiveness and endurance, helping them to form protective cell clusters and confer resistance to shear stress (Osmulski et al., 2021). Thus, understanding the mechanism of direct interaction and molecular fusion between CTCs and macrophages may help to identify therapeutic targets.

Platelet recruitment and activation also play an important role in cancer metastasis because they promote CTC survival, seeding and proliferation at secondary sites (Figure 4C) (Schlesinger, 2018; Anvari et al., 2021). CTCs form bonds with platelets to survive shear forces and mechanical stress-induced cell death in the blood stream (Labelle and Hynes, 2012). It has been hypothesized that platelets shield CTCs from mechanical stress and enhance resistance to anoikis, which is mediated via YAP1 activation (Haemmerle et al., 2017). Platelets can induce several factors (e.g., platelet-derived growth factors) that stimulate and accelerate EMT in CTCs to promote invasion and metastasis (Labelle et al., 2011; Guo et al., 2019). Platelets also help CTCs to avoid natural killer cell-mediated cytolysis by forming tumor cell-platelet micro-aggregates and generating platelet-derived normal MHC-I to the surface to escape natural killer cell recognition (Placke et al., 2012). Besides safeguarding CTCs within the bloodstream, platelets are involved in their adhesion to endothelial cells. The attachment of platelets and CTCs to the endothelial wall is mediated by platelet adhesion receptors, such as integrin αIIbβ3 and P-selectin (Coupland et al., 2012; Lonsdorf et al., 2012). A study showed that the interplay between integrin α6β1 on platelets and its receptor, a disintegrin, and metalloprotease 9 on CTCs is necessary for cancer cell extravasation (Mammadova-Bach et al., 2016). Altogether, the close and complex crosstalk between CTCs and platelets might involve distinct molecules and signaling pathways and might represent a promising antitumor strategy.

## 7 *In vitro* models mimicking cell entrapment in the microcirculation

Studying CTC behavior in the microcirculation using models that mimic the real conditions is particularly challenging. Constricted microfluidic channels with a width smaller than the cell diameter can be produced using standard microfabrication techniques, and provide an environment that mimics the blood capillaries (Irimia and Toner, 2009; Zheng and Sun, 2011; Mak et al., 2013; Portillo-Lara and Annabi, 2016; Huang et al., 2017). Higher throughput microfluidic techniques are now used to assess the mechanical characteristics of cells.

**FIGURE 5:**
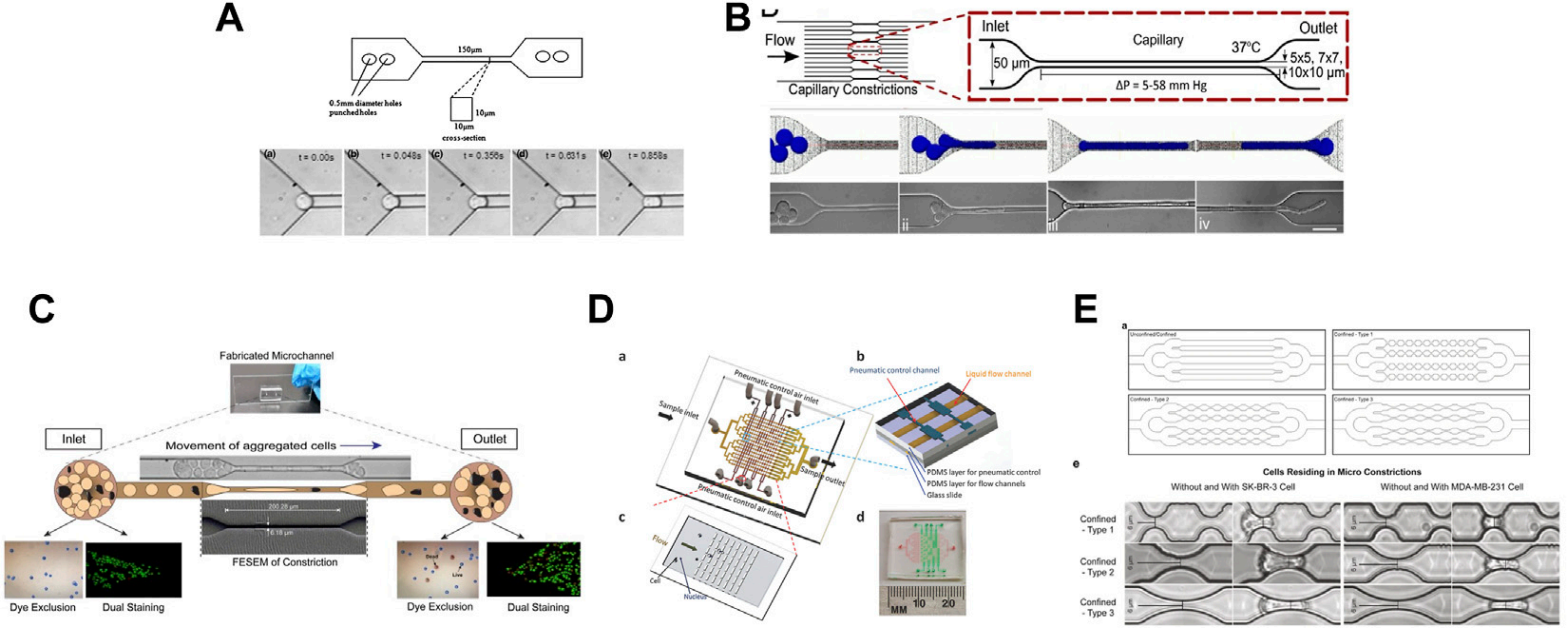
Different *in vitro* microfluidic systems that mimic the effect of the microvasculature geometry on cells. **(A)** Figure from (Hou et al., 2009). Illustration of the bonded polydimethylsiloxane (PDMS) microchannel (150 μm in length, square cross-section area of 10 by 10 μm) and optical microscopy images showing the entry of a single MCF-7 cell into a 10 by 10 μm microchannel. **(B)** Figure from (Au et al., 2016). Schematic description of a microfluidic device (16 parallel microchannels of 5 × 5, 7 × 7, or 10 × 10-µm square cross-sections) designed to mimic the capillary flow conditions and computational simulation. Micrographs show a four-cell LNCaP cluster in transit through a 5-µm capillary constriction. **(C)** Figure from (Nath et al., 2018). Schematic representation of the motion of aggregated HeLa cells passing through a microcapillary device. **(D)** Figure from (Xia et al., 2018) showing: **(A)** the design of a micro-constriction array with sixteen flow channels (in yellow) and the pneumatic control channels (in brown); **(B)**, a small portion of the pneumatic control part of the device made of three layers: PDMS layer for pneumatic control, PDMS layer for flow channels, and glass substrate; **(C)** a single patch of the micro-constriction array; and **(D)** photograph of the fabricated microfluidic device including the fluidic and pneumatic channels. **(E)** Figure from (Cognart et al., 2020). Schematic designs of microfluidic channels (upper panels) and brightfield images of SK-BR-3 and MDA-MB-231 cells before and while residing in the three types of micro-constrictions at a constant applied pressure of 10 kPa (lower panels).

Several studies have used microfluidic systems to investigate the effect of the microvasculature geometry on cells (Figure 5). For instance, constricted channels have been used to evaluate the mechanical properties of red blood cells (Shelby et al., 2003; Lee et al., 2007), leukocytes (Rosenbluth et al., 2008; Gabriele et al., 2010), and cancer cells (Raj et al., 2017). Hou et al. described a simple microfluidic channel to determine the difference in stiffness between benign and cancer breast cells (Hou et al., 2009). Khan *et al.*, demonstrated that the cell entry time into a confined space (11 μm-diameter microchannels) is a better marker of malignancy than cell deformability using glioblastoma and normal glial cell lines (Khan and Vanapalli, 2013). Au *et al.* showed that CTC clusters might rearrange reversibly to traverse 5–10 μm-wide microchannels (Au et al., 2016). Nath *et al.* made HeLa cells circulate through 7-μm-wide constrictions and found that cell viability was reduced by 50%. Conversely, expression of MMP2, a metalloproteinase involved in stromal tissue degradation, was unchanged (Nath et al., 2018). Xia *et al.* made leucocytes, MDA-MB-231 and MCF-7 breast cancer cells circulate into arrays of pores and showed that the deformation of cells and nuclei was pressure-dependent. They proposed that such studies could guide the optimization of CTC sorting devices (Xia et al., 2018). To examine the effect of different mechanical constrictions caused by geometry on the flow-driven migration of single CTCs, Cognart *et al.* constructed five microfluidic models with five distinct geometries (Cognart et al., 2020). By separately adjusting the pressure, they streamed two metastatic breast cancer cell lines, one mesenchymal-like (MDA-MB-231) and one epithelial-like (SK-BR-3), into the microfluidic device. In all models, MDA-MB-231 cells were more deformable than SK-BR-3 cells (Cognart et al., 2020). For more information, Ma *et al.* reviewed the studies that employed a microfluidic devices to examine the behavior of cancer cells during metastasis and microcirculation (Ma et al., 2018).

Microfluidic-based deformability methods have some advantages, such as easy to use, low cost and high throughput. Therefore, they are becoming a popular tool to study the cell deformability in physiological and pathological conditions. Microfluidic methods also have some limitations, because they cannot properly simulate the actual human blood capillaries and microcirculation.

Several microfluidic technologies have been developed for CTC isolation, such as Parsortix^®^ (ANGLE plc, Surrey, UK) (Miller et al., 2018), ClearCell^®^ FX1 (Biolidics Limited, Mapex, Singapore) (Lee et al., 2018), and VTX-1 (Vortex Biosciences, Menlo Park, CA, United States) (Sollier-Christen et al., 2018). These technologies are based on the differences in size, deformability and stiffness of CTCs compared with normal blood cells. They also allow studying other physical properties of CTCs, such as density and electrical charges. Li *et al.* recently provided an overview of all applications of microfluidic devices for CTC isolation and detection (Li et al., 2022).

## 8 Conclusion

Considering that metastasis is the most important cause of death in patients with cancer and that CTCs play a key role in the metastatic cascade, a deeper understanding of CTC biological properties and survival strategies in the blood circulation is essential to develop innovative clinical strategies. Using the Drake equation, it was discovered that the survival of CTCs is one of the key factors in the metastatic cascade, implying that treatments that reduce CTC survival in the vascular system could greatly reduce the development of metastasis (Dujon et al., 2021). Hence, anti-cancer therapies to target specifically CTC survival in the circulatory system should significantly reduce the risk of clinical metastases (Dujon et al., 2021). Indeed, body fluids and the associated mechanics, such as hemodynamic forces and blood vessel constriction, strongly influence the fate of tumor cells in transit and of tumor-associated factors during the metastatic process. However, multiple challenges remain to fully elucidate the involvement of the body fluid mechanics in the metastatic cascade.

At the intravasation step of the metastatic cascade, CTCs and/or CTC clusters gain access to the blood circulation and face FSS and microcirculation, where they can lodge and effectively extravasate or squeeze through and move to a more distant capillary bed where extravasation may be more convenient. The mechanical stresses created by the blood flow and capillary bed constriction forces are expected to have many different effects on CTCs. These stresses typically alter cell integrity, but they can also give to CTCs a survival advantage that may increase their capacity for metastatic spread. Moreover, heterotypic clusters increase CTC survival by helping them to avoid the physical strain of fluids, anoikis and immunological cytotoxicity. More investigations to characterize the cell behavior before and during capillary transit are urgently needed. These studies should include dynamic cell analyses to measure gene expression and EMT activation to promote more metastatic phenotypes. Cell heterogeneity also should be better investigated. To examine CTC transit or cell migration in a constrained environment, most *in vitro* studies use cell lines that do not accurately reflect or recreate CTC variability (i.e., single cells or clusters in the circulation). Therefore, we think that permanent CTC lines, such as the colon cancer-derived CTC lines established by our laboratory (Cayrefourcq et al., 2015; Soler et al., 2018) and breast cancer-derived CTC lines (Koch et al., 2020), should be used with microfluidic platforms. These microchips can mimic the capillary constriction geometries and implement physiological fluid flows. We also anticipate that mRNA, cell signaling and proteome quantification, using high-throughput technologies to assess large numbers of cells, will help to incorporate and characterize this heterogeneity and contribute to better understand how CTCs with different phenotypes may react in constrained microenvironments.
